# Smartwatch‐Derived SpO_2_ Trends During Disease Worsening and Treatment Response in Fibrotic Hypersensitivity Pneumonitis: A Case Report

**DOI:** 10.1002/rcr2.70670

**Published:** 2026-07-08

**Authors:** Tomoyuki Takahashi, Atsushi Saito, Masayuki Koyama, Kenichi Hirota, Hirofumi Ohnishi, Hirofumi Chiba

**Affiliations:** ^1^ Department of Respiratory Medicine and Allergology School of Medicine, Sapporo Medical University Sapporo Hokkaido Japan; ^2^ Department of Applied Informatics, Center for Medical Education Sapporo Medical University Sapporo Hokkaido Japan; ^3^ Division of Public Health, Department of Social Medicine School of Medicine, Sapporo Medical University Sapporo Hokkaido Japan; ^4^ Scholarly Communication Center Planning and Development Office Sapporo Medical University Sapporo Hokkaido Japan

**Keywords:** Apple Watch, interstitial lung disease, oxygen saturation, pulse oximetry, smartwatch

## Abstract

Consumer smartwatches can provide longitudinal peripheral oxygen saturation (SpO_2_) estimates for wellness purposes, but there is limited evidence that they can be used to monitor respiratory disease. In this study, we described a 52‐year‐old man with fibrotic hypersensitivity pneumonitis who developed exertional hypoxemia, which progressed radiologically and led to functional decline. Systemic corticosteroids improved his symptoms, imaging, and lung function. The monthly mean Apple Watch SpO_2_ dropped from 95.7% to 94.4% within 3 months of the worsening and recovered to 96.5% 4 weeks after corticosteroid initiation. Daily‐life SpO_2_ trends from smartwatches may be useful for monitoring respiratory diseases.

## Graphical abstract

In fibrotic hypersensitivity pneumonitis, Apple Watch‐derived SpO_2_ declined before clinical worsening and improved after corticosteroid treatment. Daily‐life smartwatch SpO_2_ trends may provide adjunctive longitudinal information for monitoring respiratory disease activity.

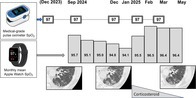

## 1 Introduction

Interstitial lung diseases (ILDs) are typically assessed using symptoms, imaging findings, pulmonary function tests and serum biomarkers during intermittent clinic visits. Wearable devices allow for the frequent collection of physiologic data in daily life, which may supplement existing assessments [1]. However, smartwatch‐derived peripheral oxygen saturation (SpO_2_) estimates are designed for wellness purposes and have not been validated for diagnostic or therapeutic decision‐making [2]. The clinical significance of longitudinal smartwatch‐derived SpO_2_ trends in ILDs is also unclear. In this study, we described a patient with fibrotic hypersensitivity pneumonitis (fHP) whose smartwatch‐derived SpO_2_ trends corresponded to disease progression and response to systemic corticosteroid therapy.

## 2 Case Report

A 52‐year‐old man developed chronic cough in 2010, and his symptoms worsened in 2020, at which point his interstitial lung abnormalities were discovered, and he was referred to our facility. He was a former smoker with a 39–pack‐year history and had no known antigen exposure. Bronchoalveolar lavage in 2021 revealed lymphocytosis (62.5%; CD4/CD8 ratio, 0.8), while transbronchial lung cryobiopsy in January 2025 revealed bronchiolocentric fibrosis, multinucleated giant cells with cholesterol clefts and lymphocytic infiltration, confirming the diagnosis of fHP [3].

In December 2024, he did not report dyspnea but recorded exertional SpO_2_ values in the 80% range on his Apple Watch, whereas resting SpO_2_ measured by a medical‐grade pulse oximeter remained stable at 97% throughout this period. Concomitantly, chest computed tomography revealed deterioration, with increased ground‐glass opacities, primarily in the left lower lobe. Pulmonary function tests revealed an objective decline within 1 year, with %VC (vital capacity predicted) falling from 49.3% to 42.3% and %DLco (diffusing capacity of the lung for carbon monoxide predicted) decreasing from 61.2% to 42.3%. Laboratory testing revealed no alternative conditions, such as infection, heart failure, thromboembolism or autoimmune disease. Krebs von den Lungen‐6 (KL‐6) increased from 1316 to 1864 U/mL, while surfactant protein‐D (SP‐D) increased from 145 to 217 ng/mL over the previous 3 months.

**FIGURE 1:**
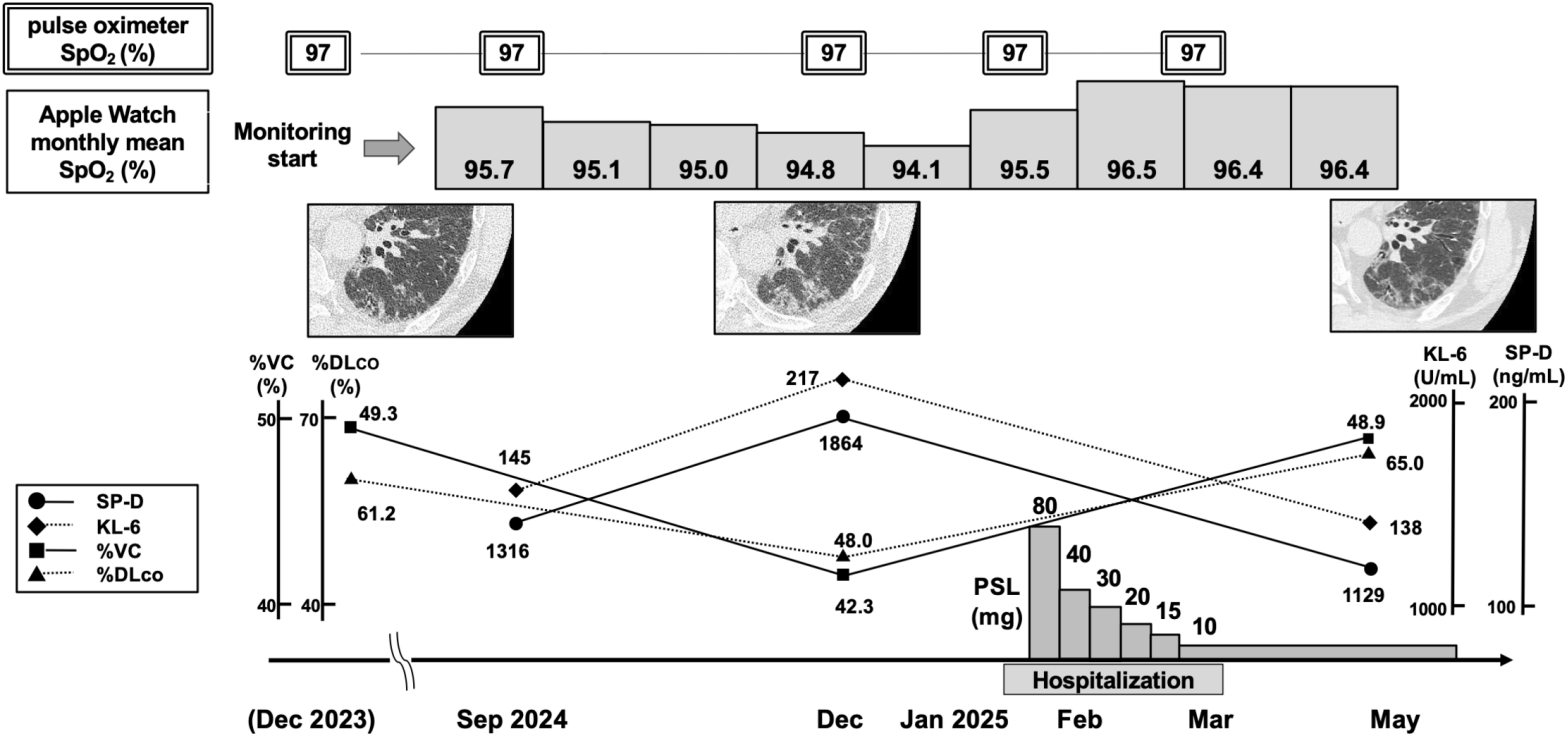
Clinical course and Apple Watch‐derived oxygen saturation trends. Monthly mean SpO_2_ values were derived from the Portable Karte health record application (Fujitsu Healthy Living Platform), linked to the Apple Health application. Representative HRCT images obtained at the indicated time points are shown at the same axial level with identical window settings. Pulmonary function (%VC and %DLco) and serum biomarkers (KL‐6 and SP‐D) are plotted at available time points. The timing and dose of systemic corticosteroid therapy are indicated along the timeline %VC, vital capacity predicted; DLco, diffusing capacity of the lung for carbon monoxide; HRCT, high‐resolution computed tomography; KL‐6, Krebs von den Lungen‐6; PSL, prednisolone.; SP‐D, surfactant protein‐D; SpO_2_, peripheral oxygen saturation.

He was diagnosed with worsening fHP and admitted in January 2025 to begin systemic corticosteroid therapy with prednisolone 80 mg/day, gradually tapering to 10 mg/day by early April 2025. Figure 1 shows that after treatment, exertional hypoxemia and radiologic abnormalities improved, as did pulmonary function and serum biomarkers.

Smartwatch monitoring began in September 2024 with an Apple Watch Series 8; the device recorded SpO_2_ levels via wrist photoplethysmography at regular intervals throughout the day and night, primarily while resting. During the observation period, an average of 20 daily SpO_2_ readings were obtained. Monthly mean SpO_2_ values, which were extracted using the Portable Karte health record application (Fujitsu Healthy Living Platform; Fujitsu Ltd., Tokyo, Japan), which is linked to the Apple Health application, decreased from 95.7% in September 2024 to 94.4% in December 2024, before increasing to 96.5% 4 weeks after corticosteroid initiation.

## 3 Discussion

This case suggests that SpO_2_ trends obtained from smartwatches during daily life may reflect disease activity and treatment response in patients with fHP. To our knowledge, no previous case report has shown parallel changes in longitudinal smartwatch‐derived SpO_2_ trends, imaging findings, pulmonary function and serum biomarkers in fHP. This case showed a longitudinal SpO_2_ trend that correlated with radiologic progression, physiological decline, biomarker changes and subsequent treatment response rather than a single abnormal smartwatch‐derived SpO_2_ value. Notably, the patient reported no dyspnea and resting SpO_2_ measured by a medical‐grade pulse oximeter remained normal. However, the smartwatch recorded exertional desaturation and the monthly mean SpO_2_ decreased during the period of clinical worsening. These findings imply that passive monitoring may capture contextual information not readily obtained during brief outpatient assessments.

Validation studies indicate that Apple Watch SpO_2_ readings are in reasonable agreement with those from medical‐grade pulse oximeters, even in patients with chronic lung disease [4, 5]. However, because these wellness‐oriented devices rely on wrist‐based photoplethysmography and proprietary algorithms, their readings may be influenced by motion, wrist positioning and peripheral perfusion. Consequently, clinically significant abnormalities should be confirmed using medical‐grade measurements [2, 4, 5]. A practical advantage of consumer wearables is their ability to obtain frequent, repeated measurements with minimal patient burden, capturing long‐term trends during clinical worsening and recovery, particularly when outpatient assessments are intermittent [1]. Although smartwatch‐derived SpO_2_ data alone cannot identify the causative antigen, combining these longitudinal trends with contextual information, such as activity records or GPS‐based location data, may help identify environments associated with disease worsening.

This report has several limitations. First, it describes a single patient, limiting the generalizability of the findings. Second, contemporaneous paired measurements with medical‐grade pulse oximetry or arterial blood gas were not systematically performed, precluding direct validation of the observed SpO_2_ changes. Third, the 1.3 percentage points in monthly mean SpO_2_ over the 3 months preceding fHP worsening fell within the reported range of intermeasurement variability for the Apple Watch among healthy users (mean difference of 2.35%, range 0%–6%) [4]; thus, the clinical significance of this change cannot be established from a single case. Finally, data were summarized as monthly averages; measurement frequency and context may have varied across months, and alternative aggregation methods might yield different results.

Future research is warranted to determine whether, under standardized measurement protocols, smartwatch‐derived SpO_2_ trends, when combined with other wearable signals and clinical measures, provide clinically meaningful information for ILD monitoring.

In conclusion, in this patient with fHP, daily trends paralleled disease worsening and treatment response. Although these values cannot substitute for medical‐grade oximetry, longitudinal trends may offer adjunctive information for ILDs monitoring.

## Author Contributions

T.T. contributed to data collection, interpretation, and drafting of the manuscript. A.S. conceived the study and supervised the work. M.K., K.H., H.O., and H.C. provided academic supervision and critically revised the manuscript.

## Funding

The author(s) declared that financial support was received for this work and/or its publication. This research was supported by an educational research grant from Sapporo Medical University, and by JSPS KAKENHI Grant Number 26K11100.

## Ethics Statement

Ethical approval was not required for this case report in accordance with the institutional policy. The manuscript was prepared following the principles of the Declaration of Helsinki.

## Consent

The authors declare that written informed consent was obtained for the publication of this manuscript and accompanying images and attest that the form used to obtain consent from the patient(s) complies with the Journal requirements as outlined in the author guidelines.

## Conflicts of Interest

The authors declare no conflicts of interest.

## Data Availability

The data that support the findings of this study are available from the corresponding author upon reasonable request.
